# Is Posttraumatic Growth Helpful in Overcoming Mental Health Disorders Due to COVID-19?: The Moderating Effect of Posttraumatic Growth in the Relationship Between COVID-19 and Psychological Health

**DOI:** 10.3389/fpsyg.2021.773326

**Published:** 2021-12-09

**Authors:** Se-Ran Park, Sun-Young Im

**Affiliations:** ^1^Department of Counseling Psychology, Seoul Digital University, Seoul, South Korea; ^2^Department of Psychology, Hallym University, Chuncheon-si, South Korea

**Keywords:** COVID-19, posttraumatic growth, depression, anxiety, committed action

## Abstract

The aim of this study was to investigate the effects of restrictions and concerns related to the coronavirus disease 2019 (COVID-19) on depression, anxiety, and committed action, and examine whether posttraumatic growth (PTG) serves as a protective factor for mental health. In particular, this study evaluated the moderating effects of PTG on the changes in levels of anxiety, depression, and committed action according to changes in COVID-19-related restrictions and concerns using a short-term longitudinal design. The Posttraumatic Growth Inventory was administered to 100 adults with significant traumatic experience living in the Republic of Korea, and the participants were asked to complete diary questionnaires on anxiety, depression, committed action, and restrictions and concerns pertaining to COVID-19. The results showed that anxiety and depression decreased, and committed action increased with an increase in PTG. These results highlight a complex relationship between restrictions and concerns related to COVID-19 and psychological health; based on the results, we discussed the positive impact of PTG on psychological health.

## Introduction

The coronavirus disease (COVID-19) has been posing a significant threat to public health worldwide since the end of 2019. The World Health Organization (WHO) declared the disease a pandemic, and owing to the spread of variants, rigorous and prolonged social distancing practices have been imposed across nations. COVID-19 not only causes concerns regarding infection among individuals and those around them, but the implementation of social distancing rules has introduced tight restrictions on many activities, such as those related to work, leisure, school, social gatherings, and religious activities (Ko, 2020; Orsolini et al., 2020). Schools have been closed and school-age children are having to stay home due to strict social distancing measures in response to COVID-19, further aggravating parents’ burden of educating and parenting their children at home (Wang et al., 2020). As a result, many people have been suffering from fear of infection as well as psychological distress, such as frustration over job loss, financial hardship, and depression and helplessness due to alienation and social distancing (Wang et al., 2020). This study was conducted from November 2020 to July 2021. Until the beginning of the study, Korea had effectively controlled the spread of the infection with a strong Korean quarantine (which is called K-Quarantine just like K-POP). However, the K-Quarantine was loosened as COVID-19 prolonged. This led to the third wave of the virus infections in November 2020 (The Korea Herald, 2020). It took more than 80 days for the country to see its virus caseload increase from 20,000 to 30,000. However, it only took 20 days for the country to add another 10,000 cases after its tally surpassed 30,000 in November 2020. Unlike the first and second waves, the cluster infection continued in an unspecified group. Consequently, social distancing was strengthened, such as only take-out possible at cafes, and “Corona Red,” that is, anger and frustration, grew beyond “Corona Blue.”

Novel infectious diseases are known to induce extreme, uncontrollable, and extensive distress beyond routine levels of stress (Main et al., 2011). People who contracted severe acute respiratory syndrome (SARS) in 2003 and survived have been reported to experience an array of mental health problems since their recovery (Cheng, 2004; Mak et al., 2009; Wing and Leung, 2012). Further, more than 65% of patients who contracted Middle East Respiratory Syndrome (MERS) in 2015 were reported to have suffered from depression, anxiety, and sleep disorders as well as posttraumatic stress symptoms even 12 months after recovery (Shin et al., 2019). According to the survey of MERS victims conducted by Lee et al. (2016), more than 90% of the Korean population felt a fear of infection for themselves and their families, and the majority of the population suffered from emotional distress. In particular, restrictions on leaving their houses increased people’s distress. Parents of young children were at 1.8 times the risk of developing a fear of infection (Nickell et al., 2004).

What factors serve as a buffer against the adverse effects of COVID-19-related restrictions and concerns on mental health? In this study, we focus on posttraumatic growth (PTG), a coping mechanism developed following trauma experienced in the past, in examining receptive attitudes and flexible responses to stressful situations related to COVID-19. The term PTG refers to the phenomenon in which humans mature and show positive changes as they encounter various crises and adversities (Tedeschi and Calhoun, 1996). People with high PTG are expected to engage in committed action, whereby they accept their situation when faced with challenges in life, establish goals commensurate with their values, and move forward, step by step, to achieve those goals (Hayes, 2004).

Until the 1990s, psychological studies were primarily focused on examining the vulnerabilities, pathological symptoms, and treatments to reduce trauma in victims (Janoff-Bulman, 1992; Foa and Riggs, 1993; Keh and Choi, 1993; Ahn, 2005). With criticism of the limited, pathological perspectives on trauma, more scientific and systematic studies on the positive impact of trauma on our lives, that is, the PTG phenomenon, began being conducted since the 1990s (Joseph et al., 1993; Tedeschi and Calhoun, 1996). People who have experience PTG have not only found significant meaning to life but also strive to live life commensurate with their values (Triplett et al., 2012; Groleau et al., 2013; Im, 2017). Furthermore, people who have achieved tremendous growth following a traumatic loss have been reported to have broadened perspectives and become highly receptive to the finiteness of life and painful emotions, such as sorrow (Im, 2013). In other words, PTG is anticipated to contribute to preserving mental health by lowering the risk of progressing to a psychopathological condition by promoting the reception of discomfort and concerns caused by limitations in stressful situations, such as falling prey to COVID-19 and encouraging individuals to commit to activities that they value. For instance, Cui et al. (2021) reported that COVID-19 ward nurses experienced PTG through psychological interventions, and it is speculated that PTG enables nurses to handle high-intensity work with a positive psychological attitude by improving self-control and relieving negative emotions (Rodríguez-Rey et al., 2019; Wu et al., 2020).

According to the PTG model recently established by Tedeschi and Calhoun (Tedeschi et al., 2018), trauma dismantles one’s basic assumptions of the world and causes suffering, but it eventually serves as an “engine” of growth (Choi, 2014; Triplett et al., 2012; Eom and Cho, 2016). Individuals engage in automatic and intrusive rumination immediately after suffering from trauma, but at some point after the traumatic episode, they re-evaluate the meaning of adversity and begin to introspect, looking back (Taku et al., 2008).

Although limited in number, studies have substantiated that PTG is a real phenomenon and it serves as a protective factor against burnout. Taku (2014) proved that PTG protects surgeons against burnout. Even physicians with low social support, a factor of resilience, were found to experience maladjustment, such as depersonalization and achieve greater personal accomplishments, if they experienced significant PTG (Taku, 2014). Gibbons et al. (2011) reported that burnout is negatively associated with growth in social workers. Thus, we examined whether PTG is indeed a protective factor that reduces individuals’ levels of depression and anxiety due to COVID-19 and increases their committed action.

COVID-19 has inflicted intense psychological suffering on healthcare providers. Yang et al. (2020) reported that 85.4% of frontline nurses sustained stress responses, such as somatization and compulsive symptoms, and moderate or severe psychological suffering. They are consistently exposed to infection risk during work and consequently required to be separated from their families, which makes it difficult for them to receive familial support when in need. Korean studies have examined the suffering of nurses—healthcare providers who must deal with patients in closed spaces for prolonged periods. Park (2018) reported that stress in nurses is aggravated by their having to remain in an isolation ward with a patient while wearing personal protection equipment for more than 90 min at a time and having to perform tasks, such as cleaning, in addition to nursing work due to the closed access to isolation wards for non-qualified personnel. High job stress and accumulated fatigue in such circumstances lead to burnout (Salari et al., 2020; Jeong, 2021). Similarly, COVID-19-related restrictions and concerns are believed to induce depression and anxiety.

Social restrictions and concerns pertinent to COVID-19 may thwart value-based committed action by reducing individuals’ vigor and causing a sense of isolation. Committed action is conceptualized as that being a result of “free choice and voluntariness from intrinsic motivation” (Wilson and DuFrene, 2009), and voluntariness and freedom not only increase vigor by providing reinforcements, such as pleasure, but also contribute to psychological wellbeing by conserving energy through self-regulatory control (Wilson and Sandoz, 2008). If individuals cannot adopt engagement response commensurate with their values due to restrictions, such as those imposed during the COVID-19 pandemic, the risk for persistence or exacerbation of depression is elevated (Zettler, 2007; Wilson et al., 2010). Moreover, some argue that individuals who are overwhelmed by anxiety lose their values and cannot live an engaged life because their views become limited to simply focusing on the imminent threat (Eifert and Forsyth, 2005). An engaged, pleasant, and meaningful life, is one aspect of happiness proposed by Seligman (2002).

On the other hand, restrictions and concerns pertinent to COVID-19 may actually promote committed action. Committed action refers to discovering values and living life in accordance with those values, and this is similar to the discovery of meaning in life. Although few studies have substantiated the relationship between committed action and COVID-19, many studies that investigated the association between meaning in life and COVID-19 observed that individuals discover life to be more meaningful during the COVID-19 pandemic than before it. In other words, amid the demands to restructure daily lives during the COVID-pandemic, people are likely to think about the meaning of their lives more than before and engage in relevant activities (De Jong et al., 2020; Eisenbeck et al., 2021), and it has been reported that people have discovered more meaning in life (Yang et al., 2021). Thus, it is necessary to explore the impact of COVID-19-related restrictions and concerns on committed action.

To secure ecological validity, this study used a diary method, a type of Ecological Moment Assessment (EMA). Diary research is advantageous as it (1) considers naturalistic settings compared to a cross-sectional study design, (2) avoids possible retroactive contamination by investigating ongoing experiences, (3) considers fluctuation among individuals, such as personality, mood, and behavior, while a cross-sectional study considers variables as a fixed value, and (4) examines person–situation interaction considering the contextual effect of individual characteristics (Shiffman et al., 2008; Ohly et al., 2010; Iida et al., 2012).

The degree of mood, such as feelings like anxiety and depression, or the degree of activity, such as commitment action measured in this study, is typically a variable that fluctuates according to daily experiences (e.g., Hankin et al., 2005; Starr and Davila, 2012). In addition, as described above, it was assumed that the level of depression, anxiety, and commitment behavior would fluctuate in response to the level of COVID-19-related restrictions and concerns. The diary method was thought as appropriate to dynamically examine the interaction between changes in levels of depression, anxiety, and commitment behavior according to COVID-19-related restrictions and concerns (within factor) and PTG (between factor).

In the present study, we used a short-term longitudinal design to examine the moderating effect of PTG by collecting data on mental health-related parameters (depression, anxiety, and committed action) at several time points to ensure higher reliability of data than that measured at a single point. We established the following hypotheses: First, PTG moderates the effect of COVID-19-related restrictions and concerns on depression. Second, PTG moderates the effect of COVID-19-related restrictions and concerns on anxiety. Third, PTG moderates the effect of COVID-19-related restrictions and concerns on committed action.

## Materials and Methods

### Participants

Adults aged 20–59 years in the Republic of Korea, who has experienced trauma, were enrolled in the study. Participant recruitment and survey administration were performed through a company conducting online surveys. The basis for the sample size of 100 we used are as follows. (1) According to Maas and Hox (2005), small sample size at level 2(a sample of 50 or less) leads to biased estimates of the second-level standard error. (2) Considering that HLM is basically a regression analysis, the number of survey participants required was calculated using the G*Power 3.1.7 program. In the regression analysis, using as many independent variables as possible, it was confirmed that at least 90 people were required when the sample size was calculated by setting the Effect size (f2) to 0.2, *α* to 0.05, and power to 0.95when the sample size was calculated by setting the Effect size (f2) to 0.2, *α* to 0.05, and power to 0.95. Although 176 people enrolled in the study, data of those who completed all four diary questionnaires were finally analyzed.

The COVID-19 pandemic, triggered by the spread of an infectious disease, was considered a stressful event causing substantial fear and anxiety and forcing the enforcement of societal restrictions. A professional survey company conducting studies specified that this study was conducted on individuals with trauma experiences to recruit eligible participants. A posttraumatic experience in our study was taken to be a broader concept than the traditional definition of trauma in the DSM-5, and it was operationally defined as the experience of an event that inflicted tremendous psychological shock and pain. The usage of the term “trauma” in the PTG-related research domain is a bit broader and more inclusive than DSM-5 (American Psychiatric Association, 2013) criteria (Zoellner and Maercker, 2006). Tedeschi and his colleagues (1996, 2004, 2018) have used the terms “trauma,” “crisis,” and “major stressor” as essentially synonymous expressions in their studies. PTG has often been studied among people having a severe disease like cancer or brain injury (Kent et al., 2013; Adams, 2015; Karagiorgou and Cullen, 2016; Cheng et al., 2017) and among people experiencing loss of close and beloved ones due to bereavement or divorce (Calhoun et al., 2010; Albuquerque et al., 2018).

The data were collected from 5th to 14th April 2021, and no other special events that strongly influenced the results occurred during that period, such as a sharp increase in the number of people infected with COVID-19 or major changes in the government’s quarantine policy.

### Instruments

#### Baseline Questionnaire

##### Trauma Experience Questionnaire

To determine whether the participants had significant trauma experiences, they were first asked a question about whether they had experienced trauma in the past 10 years and then asked to specify the type of trauma and describe the details of the event. We used a modified version of the list of events used by Tedeschi and Calhoun (1996) and re-classified the types of trauma into eight types: loss of a loved one, academic and task failure, financial hardship, abuse and harsh treatment, one’s accident and injury, one’s severe disease, accident and disease of a family member or close friend, and others.

##### Posttraumatic Growth Inventory-Revision (PTGI-X)

We used the PTGI, a self-report questionnaire originally developed by Tedeschi and Calhoun (1996) to measure individuals’ perception of positive changes after a traumatic event, with four additional items about spiritual-existential change. The PTGI-X contained 25 items for five subscales: new possibilities, relating to others, personal strength, appreciation of life, and spiritual-existential change. Each item was rated on a six-point scale ranging from 0 (never experienced) to 5 (experienced a lot). The tool was reported to have good reliability and construct validity according to the study by Tedeschi et al. (2017). They reported that Cronbach’s α was 0.95–0.97. and that the measures of deliberate rumination and core belief challenge were related to the PTGI-X scores, but intrusive rumination was not, consistent with findings in the previous literature (Cann et al., 2010). The internal consistency of the study was 0.97.

#### Diary

##### Questionnaire About COVID-19-Related Restrictions and Concerns

We developed a questionnaire that comprehensively measured the psychosocial and practical restrictions imposed by the spread of COVID-19 and consequent concerns and threats with reference to a previous study (Ko, 2020). The questionnaire contained three items about “threat from COVID-19,” “perceived concerns about risks,” and “restrictions in life” rated on a scale from 1 to 5. The Cronbach’s α was 0.75 in the study by Ko (2020) and 0.73–0.81 across various time points in this study.

##### Depression

The Patient Health Questionnaire-9 (PHQ-9) developed by Spitzer et al. (1999) and adapted and validated by Park et al. (2010) was used to measure depression after modifying the items to reflect experiences over the past 3 days. This nine-item tool was rated on a scale from 1 (none) to 4 (every day). The Cronbach’s α was 0.81 in the study by Park et al. (2010) and 0.88–0.90 across various time points in this study.

##### Anxiety

The Generalized Anxiety Diorder-7 (GAD-7) developed by Spitzer et al. (2006) and adapted and validated by Ahn et al. (2019) was used to measure anxiety after modifying the items to reflect experiences in the past 3 days. This seven-item tool was rated on a scale from 1 (not bothered at all) to 4 (bothered every day). The Cronbach’s α was 0.93 in the study by Ahn et al. (2019) and 0.93–0.95 across various time points in this study.

##### Committed Action

The Engaged Living Scale (ELS) developed by Trompetter et al. (2013) and adapted and validated by Park (2020) was used to measure committed action after modifying the items to reflect experiences in the past 3 days. The scale comprised two subscales, “Valued living” and “Committed action,” and all 16 items were rated on a scale of 1 (never) to 5 (always). The α was 0.90 in the study by Trompetter et al. (2013) and 0.96 across all time points in our study.

### Procedure

An online survey system was used to recruit people with significant traumatic experiences nationwide and administer a baseline questionnaire after obtaining informed consent. Prior to the baseline survey, the company conducting the survey specified that, since this study was being conducted on people with traumatic experiences, only those who considered themselves eligible to participate in the study ought to enroll. The baseline questionnaire contained three items from the Trauma Experience Scale and 25 items from the PTGI-X. After completing the baseline questionnaire, the participants were asked to make a diary entry four times at three-day intervals. The diary consisted of a questionnaire for COVID-19-related restraints and concerns, committed action, depression, and anxiety. The baseline survey and first diary were completed on the same day, and the participants were sent an online link to the diary through social media at three-day intervals, thereafter, to complete the second, third, and fourth entries.

### Statistical Analysis

As this study was a short-term longitudinal one based on the collection of data pertaining to individuals’ daily experiences at several time points, the data were multilevel, with the diary data (level 1) at each time point nested in individuals (level 2). Thus, we used hierarchical linear modeling (HLM), which is appropriate for the analysis of multilevel data (Singer et al., 2003). The restricted maximum likelihood approach was used on the HLM 8.0 (Scientific Software International, Lincolnwood, Inc., United States) for estimation. Level 1 intrapersonal variables were group-mean centered, while level 2 interpersonal variables were grand-mean centered.

To test the study’s hypothesis, we first tested whether COVID-19-related restrictions and concerns (CoF*_ti_*) affect depression, anxiety, and committed action at level 1. Thereafter, to examine the inter-level interactions, PTG (PTG*_i_*) was entered at level 2. Further, because time itself is not theoretically assumed to affect the change in the dependent variable at level 1, we did not consider time as a variable. The equations for the model at each level were as follows.

- Level 1 model

Depression:


$$D_{ti}=\pi_{0i}+\pi_{1i}\times \left({\mathrm{CoF}}_{ti}\right)+e_{ti}$$

Anxiety:


$$A_{ti}=\pi_{0i}+\pi_{1i}\left({\mathrm{CoF}}_{ti}\right)+e_{ti}$$

Committed action:


$${\mathrm{CA}}_{ti}=\pi_{0i}+\pi_{1i}\times \left({\mathrm{CoF}}_{ti}\right)+e_{ti}$$

- Level 2 model

$$\pi_{0i}=\beta_{00}+\beta_{01}\times \left({\mathrm{PTG}}_{i}\right)+r_{0i}$$

$$\pi_{1i}=\beta_{10}+\beta_{11}\times \left({\mathrm{PTG}}_{i}\right)+r_{1i}$$

Here, *π*_0*i*_ is the baseline value for each individual, that is, average of committed action, acceptance, depression, and anxiety for the response to the COVID-19-related restrictions and concerns; *π_1i_* is the rate of change, that is, the response to the COVID-19-related restrictions and concerns in terms of depression, anxiety, and committed action on a particular day.

## Results

### Demographic Characteristics and Types of Traumatic Events

Of the 100 participants, 50 were men and 50 women. The mean age was 39.9 years (SD = 11.43). Fifty were single, and 50 were married. Fifty-one had a child, while 49 did not. Sixty-two did not adhere to a religion, while 38 did. The most common traumatic event that had the most significant impact was loss of a loved one (*n* = 34), followed by accidents and diseases of a family member or close friend (*n* = 22), academic and task failure (*n* = 19), financial hardship (*n* = 10), severe disease (*n* = 6), accident and injury (*n* = 4), abuse and harsh treatment (*n* = 4), and others (*n* = 1) (Table 1).

**Table 1:** Demographic characteristics of Korean participants (*n* = 100).

Demographic Variable	*n* (%)	Demographic Variable	*n* (%)
Gender	Religion
Men	50 (50.0%)	Christian	19 (19.0%)
Women	50 (50.0%)	Catholic	9 (9.0%)
Age Groups		Buddhist	5 (5.0%)
20s	24 (24.0%)	Protestantism	2 (2.0%)
30s	21 (21.0%)	Won Buddhism.	2 (2.0%)
40s	26(26.0%)	Oriental philosophy	1 (1.0%)
50s	29 (26.0%)	No Religion	62 (62.0%)
Marital status		Trauma Type	
Married	50 (50.0%)	Loss of a Loved One	34 (34.0%)
Single	44 (44.0%)	Accidents and Disease of Close Acquaintance	22 (22.0%)
Divorced or Separated	5 (5.0%)	Academic Failure	19 (19.0%)
Others	1 (1.0%)	Financial Hardship	10 (10.0%)
Parental status		Severe Disease	6 (6.0%)
Have Children	51 (51.0%)	Accident and Injury	4 (4.0%)
Do Not Have Children	49 (49.0%)	Abuse and Harsh Treatment	4 (4.0%)
Job		Others	1 (1.0%)
Service	14 (14.0%)		
Office Worker	44 (44.0%)		
Engineer	7 (7.0%)		
Others	35 (35.0%)		

The effects of demographic variables of gender, age, marital status, child presence, occupation, and religion on depression, anxiety, and committed action were analyzed through HLM. As a result, only the effect of the presence or absence of children on the committed action was significant, and the analysis was conducted by including the presence or absence of children in the analysis using future committed action as a dependent variable.

### Depression

In the null model, there was a significant difference in depression between individuals at the intercept (*p* < 0.001). Furthermore, the intraclass correlation coefficient (ICC) was 77%, with level 2 variance accounting for a high percentage of the total variance, confirming the need to use a multilevel model. Thereafter, the values of the outcome variables were entered in level 1 to examine the effects of COVID-19-related restrictions and concerns on depression (Table 2), and the regression coefficient of the slope was not significant. However, there were significant differences among the baseline values, so the factors that differed between individuals could be analyzed at level 2. In addition, the rate of change of committed action as a result of COVID-19-related restrictions and concerns increased with increasing baseline values.

**Table 2:** Effects of COVID-19-related restrictions and concerns on depression.

Fixed effect	Coefficient	Standard error	*t*	*df*	*p*
Intercept 1, *π*_0_					
Intercept 2, *β*_00_	16.28	0.56	29.06	98	<0.001
CoF slope, *π*_1_					
Intercept 2, *β*_10_	0.25	0.13	1.98	98	0.05
Random effect	Standard deviation	Variance	*df*	χ^2^	*p*
Intercept 1, *r*_0_	5.39	29.09	88	1384.73	<0.001
CoF slope, *r*_1_	0.52	0.27	88	118.17	0.02
Level 1, e	2.82	7.95			

*CoF = COVID-19-related restrictions and concerns*.

#### Effects of PTG on the Relationship Between COVID-19-Related Restrictions and Depression

Since the random effect was significant at *r*_0_ and *r*_1_ in an unconditional growth model, PTG, the independent variable at level 2 that could explain the individual differences, was added to analyze the baseline values and individual differences (Table 3). PTG had a negative effect on the baseline depression. In other words, people with greater PTG had lower levels of depression. The slope and intercept for changes in levels of depression according to COVID-19-related restrictions and concerns were not significant, and PTG did not have a significant effect on the slope representing change. In other words, levels of depression remained similar to individuals’ baseline values, regardless of COVID-19-related restrictions and concerns.

**Table 3:** The moderating effect of PTG in the effect of COVID-19-related restrictions and concerns on depression.

Fixed effect	Coefficient	Standard error	*t*	*df*	*p*
Intercept 1, *π*_0_					
Intercept 2, *β*_00_	16.28	0.54	29.89	97	<0.001
PTG, *β*_01_	−0.05	0.02	−2.59	97	0.01
CoF slope, *π*_1_					
Intercept 2, *β*_10_	0.25	0.13	1.98	97	0.05
PTG, *β*_12_	0.00	0.00	0.10	97	0.92
Random effect	Standard deviation	Variance	*df*	χ^2^	*p*
Intercept 1, *r*_0_	5.23	27.38	87	1291.27	<0.001
CoF slope, *r*_1_	0.52	0.27	87	117.79	0.02
Level 1, e	2.82	7.97			

*CoF = COVID-19-related restrictions and concerns*.

### Anxiety

There was a significant difference in anxiety between individuals at the intercept (*p* < 0.001). Further, the ICC was 74%, with level 2 variance accounting for a high percentage of the total variance, confirming the need to use a multilevel model. The values of the outcome variables were entered in level 1 to examine the effects of COVID-19-related restrictions and concerns on anxiety (Table 4), and the regression coefficient of the slope was not significant, showing that anxiety did not change according to COVID-19-related restrictions and concerns. Additionally, the variance of the slope for changes in anxiety according to COVID-19-related restrictions and concerns was significant, and the rate of change of anxiety according to COVID-19-related restrictions and concerns increased with increasing values of baseline anxiety.

**Table 4:** Effects of COVID-19-related restrictions and concerns on anxiety.

Fixed effect	Coefficient	Standard error	*t*	*df*	*p*
Intercept 1, *π*_0_					
Intercept 2, *β*_00_	12.48	0.50	24.88	98	<0.001
CoF slope, *π*_1_					
Intercept 2, *β*_10_	0.24	0.12	1.92	98	0.06
Random effect	Standard deviation	Variance	*df*	χ^2^	*p*
Intercept 1, *r*_0_	4.82	23.19	88	1284.93	<0.001
CoF slope, *r*_1_	0.54	0.29	88	110.69	0.05
Level 1, e	2.65	7.02			

*CoF = COVID-19-related restrictions and concerns*.

#### Effects of PTG on the Relationship Between COVID-19-Related Restrictions and Anxiety

Because the random effect was significant at *r*_0_ and *r*_1_ in an unconditional growth model, PTG, the independent variable at level 2 that could explain the individual differences, was added to analyze the baseline values and individual differences (Table 5). Results showed that PTG had a negative effect on the baseline anxiety. In other words, people with greater PTG had lower levels of anxiety. The slope and intercept for changes in anxiety according to COVID-19-related restrictions and concerns were not significant, and PTG did not have a significant effect on the slope representing change.

**Table 5:** The moderating effect of PTG in the effect of COVID-19-related restrictions and concerns on anxiety.

Fixed effect	Coefficient	Standard error	*t*	*df*	*p*
Intercept 1, *π*_0_					
Intercept 2, *β*_00_	12.48	0.49	25.49	97	<0.001
PTG, *β*_01_	−0.04	0.02	−2.46	97	0.002
CoF slope, *π*_1_					
Intercept 2, *β*_10_	0.23	0.12	1.88	97	0.06
PTG, *β*_11_	0.00	0.00	−0.44	97	0.66
Random effect	Standard deviation	Variance	*df*	χ^2^	*p*
Intercept 1, *r*_0_	4.69	21.97	87	1208.35	<0.001
CoF slope, *r*_1_	0.55	0.03	87	110.54	0.05
Level 1, e	2.65	7.01			

*CoF = COVID-19-related restrictions and concerns*.

### Committed Action

Prior to performing a multilevel modeling analysis, we analyzed an unconditional model (null model) that only contained level 1 outcome variables to check for the suitability of the data for HLM (Table 1). There was a significant difference in the level of committed action between individuals at the intercept (*p* < 0.001). Further, the ICC was 78%, with level 2 variance accounting for a high percentage of the total variance, confirming the need for a multilevel model.

Thereafter, the values of the outcome variables were entered in level 1 to examine the effects of COVID-19-related restrictions and concerns on committed action (Table 6). The regression coefficient of the slope was significant, and on average, committed action showed a tendency to increase with greater COVID-19-related restrictions and concerns. There were significant differences among the individuals’ baseline values, so the factors that differed between individuals could be analyzed at level 2. The variance of the slope for changes in committed action according to COVID-19-related restrictions and concerns was significant at a level of 0.05, and the rate of change of committed action according to COVID-19-related restrictions and concerns increased with increasing baseline values of committed action.

**Table 6:** Effects of COVID-19-related restrictions and concerns on committed action.

Fixed effect	Coefficient	Standard error	*t*	*df*	*p*
Intercept 1, *π*_0_					
Intercept 2, *β*_00_	48.47	1.30	37.19	98	<0.001
CoF slope, *π*_1_					
Intercept 2, *β*_10_	1.04	0.28	3.76	98	<0.001
Random effect	Standard deviation	Variance	*df*	*χ* ^2^	*p*
Intercept 1, *r*_0_	12.60	158.79	88	1632.88	<0.001
CoF slope, *r*_1_	1.70	1.27	88	114.64	0.03
Level 1, e	6.12	37.46			

*CoF = COVID-19-related restrictions and concerns*.

Additionally, we analyzed the effects of demographic factors, such as age, marital status, and children on committed action, and the results indicated that committed action was greater among childless individuals.

#### Effects of PTG on the Relationship Between COVID-19-Related Restrictions and Committed Action

Because the random effect was significant at *r*_0_ and *r*_1_ in the unconditional growth model, PTG, the independent variable at level 2 that can explain individuals’ differences, was added to analyze the baseline values and individuals’ differences (Table 7). It was found that PTG had a positive effect on the baseline level of committed action. In other words, people with greater PTG engaged in more committed action. The relationship between the slope and intercept for changes in committed action according to COVID-19-related restrictions and concerns was not significant. Additionally, we analyzed the effects of children and PTG together on committed action. It was found that PTG had a positive effect on the baseline level of committed action, but effect of children on committed action was not significant.

**Table 7:** The moderating effect of PTG in the effect of COVID-19-related restrictions and concerns on committed action.

Fixed effect	Coefficient	Standard error	*t*	*df*	*p*
Intercept 1, *π*_0_					
Intercept 2, *β*_00_	48.47	0.90	53.84	97	<0.001
PTG, *β*_01_	0.34	0.03	10.41	97	<0.001
CoF slope, *π*_1_					
Intercept 2, *β*_10_	1.02	0.27	3.67	97	<0.001
PTG, *β*_11_	0.01	0.01	1.22	97	0.23
Random effect	Standard deviation	Variance	*df*	χ^2^	*p*
Intercept 1, *r_0_*	8.42	70.90	87	783.71	<0.001
CoF slope, *r_1_*	1.13	1.27	87	113.08	0.03
Level 1, e	6.11	37.40			

*CoF = COVID-19-related restrictions and concerns*.

## Discussion

This study aimed to investigate how PTG acted as a buffer against the psychological impact of the restrictions and concerns related to COVID-19 using a short-term longitudinal design. More specifically, we analyzed whether people with high PTG exhibited a lower level of anxiety and depression in relation to the restrictions and concerns pertinent to COVID-19 and how PTG influenced their level of committed action. The key findings were as follows.

First, on an average, depression and anxiety were not associated with COVID-19-related restrictions and concerns. Second, committed action increased with increasing COVID-19-related restrictions and concerns. In other words, people with greater COVID-19-related restrictions and concerns actually lived their lives more commensurate with their values. Third, individuals who achieved greater levels of PTG were less depressed and less anxious. Fourth, individuals who achieved greater levels of PTG engaged in more committed action.

Contrary to our expectation that COVID-19-related restrictions and concerns would adversely impact mental health, COVID-19-related restrictions and concerns were not significantly associated with depression and anxiety. Previous studies and media coverage of infectious disease disasters have shown that quarantine experience triggers acute stress responses, such as anxiety, irritation, insomnia, and reduced concentration in healthcare workers (Bai et al., 2004), and that a suspension of routine activities and severed social interactions aggravated loneliness and frustration (Orsolini et al., 2020). Thus, we expected that people with greater actual or perceived COVID-19-related restrictions and concerns would show a higher level of depression and anxiety, but we did not observe a statistically significant association between the two. We propose a few reasons for this result. First, while social distancing practices also have negative repercussions, they can induce positive changes depending on individuals’ personality traits. For example, people who have difficulty with face-to-face activities and interactions due to tendencies, such as high avoidance might actually benefit from social distancing in terms of their mental health and life, as it ensures an adequate amount of time alone. In particular, one characteristic feature of Korean social culture is frequent mandatory corporate dinners and group activities, and the prohibition of such activities during the pandemic might have been welcomed by these individuals. However, this is a unique phenomenon observed in the strongly collectivist Korean society, so subsequent studies should compare the impact of restrictions provoked by COVID-19 on individuals’ lives and mental health. Another reason may be that this study was conducted 1 year after the outbreak of COVID-19 and, therefore, many people became accustomed to the changes in their lives due to social distancing. For this reason, it is possible that their perceived inconveniences from social distancing measures were considerably reduced regardless of COVID-19-related restrictions and concerns. In conclusion, our results suggest that the impact of COVID-19-related restrictions and concerns on people’s lives may be quite complex, depending on individuals’ traits and the time of observation.

Following this, we discuss the increased rate of committed action with increasing COVID-19-related restrictions and concerns. First, this may be attributable to the sufficient time secured for a committed action because people have strictly adhered to social distancing practices, such as avoiding social gatherings, due to COVID-19-related restrictions and concerns. Although this topic has not been exactly studied in the literature, a Dutch study reported that people have been engaging in more online leisure activities at home as well as traditional outdoor solo sport activities, such as hiking and running, since the implementation of social distancing norms due to COVID-19 (van Leeuwen et al., 2020), suggesting that people are finding more time to spend on solo leisure activities.

These results can be interpreted in relation to the result that men and childlessness were identified as the predictors of committed action according to COVID-19-related restrictions and concerns. In Korea, the duration of household labor, including caregiving labor, is 3.6 times higher among women than men (Park and Cho, 2020). Childless individuals or men spend less time on household chores or parenting compared to their parents or female counterparts, respectively. Thus, they have relatively more spare time and be more relaxed psychologically to commit to an engaged life in line with their values.

In addition to having more time to live an engaged life, people might have also been given the opportunity to reflect on and reestablish their values and practice them. An Italian study that surveyed the impact of COVID-19 on the meaning in life during a lockdown and performed a cluster analysis observed that rearranging personal priorities was one of the four key factors (Venuleo et al., 2020). Some argued that meaning can be restructured (De Jong et al., 2020) and engagement in meaningful activities increased during the process whereby daily lives were dismantled by COVID-19 are, subsequently, reestablished (Eisenbeck et al., 2021). In a three-month longitudinal study conducted in China, meaning making about negative events increased, which in turn resulted in reduced psychological distress during the COVID-19 pandemic (Yang et al., 2021), and a four-day short-term longitudinal study reported that people with a higher understanding of the meaning of life were less stressed about COVID-19 (Trzebiński et al., 2020). In light of these studies, it is possible that people have rearranged their lives’ priorities to focus on those activities that they had been postponing and pursue meaningfulness in life when given enough time alone.

The moderating effect of PTG, which was the focus of our study, was confirmed. In other words, regardless of COVID-19-related restrictions and concerns, depression and anxiety decreased on average with increasing PTG, and people who achieved greater PTG demonstrated a higher baseline level of committed action. These results suggest that PTG positively contributes to psychological health and protects against psychological distress. We experience growth in a variety of areas through crises and adversities (Tedeschi et al., 2018). In particular, personal growth, referring to the awareness of one’s potential and resilience, enhancement of their ability to cope with difficulties, and greater confidence, can be considered a factor that helps individuals protect themselves and respond flexibly to the confusion and fear around the COVID-19 pandemic. Several studies have reported that PTG is negatively correlated with psychological distress (Powell et al., 2003), and it has also been reported that PTG is negatively associated with depression and anxiety among SARS survivors (Cheng et al., 2006). In fact, PTG has also been reported to be negatively associated with psychological distress in frontline healthcare workers who battle against the COVID-19 pandemic (Cui et al., 2021). In particular, Korea adopted the most rigorous social distancing measures in response to the COVID-19 pandemic among countries worldwide (Kim et al., 2021), and it is speculated that mature emotional regulation achieved by PTG lowered the risk of progressing to psychopathologies, such as depression and anxiety, and contributed to individuals’ commitment to activities they value. The protective effects of PTG against burnout (Taku, 2014) can also be understood in line with our findings. Committed action refers to discovering values and living a life in line with these values. This is closely linked to the discovery of meaning in life. While few studies have investigated the association between committed action and PTG, many studies have supported the positive association between meaning in life and PTG (Triplett et al., 2012; Groleau et al., 2013; Im, 2017). Further, Im (2017) reported that life satisfaction increases with increasing agreement between one’s life and its meaning. Taken together, people who have achieved PTG are able to discover meaning in life better and realize their values through committed action.

This study had several limitations. First, the total duration of data collection was only 12 days; therefore, subsequent studies should ensure a sufficient duration of study to retest the hypothesis. To examine individual reactions to various phases of COVID-19 more dynamically, we think it would have been nice to measure them over a longer period of more than 6 months. Second, because of the COVID-19 situation, the online survey was the most realistic method of collecting data; however, it did not guarantee sufficient representation of the sample. In order to secure representation, an online research company with a large-scale national research panel was selected, and the ratio of demographic characteristics, such as gender and age, was tried to be maintained similar to the actual one, but offline surveys will be needed for more representative sampling. Third, the path through which COVID-19-related restrictions and concerns increases committed action needed to be explored further. In particular, studies should examine the increase in committed action through the mediation of leisure time. Fourth, other types of measurement for anxiety and depression should be taken. It is possible that we could not accurately measure the emotional changes resulting from COVID-19 because these measurements do not change over a short period of time. The instrument we used to measure COVID-19-related restrictions and concerns was a three-item scale developed by a Korean developer (Ko, 2020). COVID-19-related restrictions and concerns were not significantly associated with depression and anxiety in our study, and this may be due to the small number of items in the scale. We suggest that better instruments to measure the COVID-19-related restrictions and concerns which have enough items and higher reliability should be developed for further study.

Despite these limitations, this study has several methodological strengths and implications. First, in contrast to the predominant use of cross-sectional designs to study the association between PTG, psychological health, and meaning in life, we collected data based on daily life experiences using the diary method, which enhanced the ecological validity of our study. Second, we applied multilevel modeling to measure dynamic person-situation interactions. By analyzing the interaction of PTG with COVID-19-related restrictions and concerns, we were able to more thoroughly examine individuals’ behavioral patterns during the COVID-19 pandemic.

## Conclusion

COVID-19-related restrictions and concerns would adversely impact mental health. COVID-19-related restrictions and concerns were not significantly associated with depression and anxiety, but with the increased rate of committed action. We proposed a few possible reasons, such as social distancing practices, can induce positive changes depending on individuals’ personality traits or cultural context. And this study is an empirical one that evaluated the contribution of PTG in dealing with infectious disease disasters, where our findings provide evidence supporting the fact that qualitative changes attained by overcoming a crisis in life are beneficial for coping with future crises in and maintaining a high quality of life.

## Data Availability Statement

The raw data supporting the conclusions of this article will be made available by the authors, without undue reservation.

## Ethics Statement

The studies involving human participants were reviewed and approved by institutional review board of Hallym University. The patients/participants provided their written informed consent to participate in this study.

## Author Contributions

SI and SP jointly participated in conceptualization, data collection, and statistical analysis. In draft writing, SP and SI jointly wrote the first draft on Introduction, Discussion, Method, and Results. Afterward, the authors reviewed and edited the draft together, and approved the final draft.

## Funding

This research was supported by the Hallym University Research Fund, 2018 (HRF-201809-002).

## Conflict of Interest

The authors declare that the research was conducted in the absence of any commercial or financial relationships that could be construed as a potential conflict of interest.

## Publisher’s Note

All claims expressed in this article are solely those of the authors and do not necessarily represent those of their affiliated organizations, or those of the publisher, the editors and the reviewers. Any product that may be evaluated in this article, or claim that may be made by its manufacturer, is not guaranteed or endorsed by the publisher.
